# Case of Alveolar Exostosis

**Published:** 1841-12

**Authors:** S. M. Shepherd

**Affiliations:** Surgeon Dentist, of Petersburg, Va.


### ARTICLE VII.

*Case of Alveolar Exostosis.* By S. M. SHEPHERD, Surgeon Dentist, of Petersburg, Va.

A gentleman, about thirty-five years of age, called on me a few days since, desiring to have two artificial teeth inserted. As soon as he entered my office, I thought I perceived a want of a central incisor tooth. But upon examination, I found that the space which I had supposed was occasioned by the loss of a tooth, was between the superior central incisors, and that all the front teeth were present, including the bicuspides. The space was square and even, and presented exactly the appearance of the loss of a tooth, with this difference only, that it was in the centre of the mouth. My attention was then directed to the lower teeth, which I found precisely in the same condition. The space in this instance, was considerably wider, especially at the points of the teeth, which diverged a little, making it necessary to insert two instead of one. I have seen many cases in which the teeth have been forced apart by some unhealthy action in the gum sufficiently wide to admit of another tooth, and when the gum became healthy, the teeth retained the position into which they had been thrown; and in some cases I have inserted teeth where spaces have been thus produced; but I have never before seen a case in which the gum had always been healthy, that would at all admit of it. In some cases the removal of a supernumerary tooth in early life, leaves a space that fails to close up; and my first thought was, that such must have been the case in this instance but I was assured by the gentleman that no such removal had ever taken place.*

### *Suppression of Quackery in Canada.*

That contemptible travelling quack, Williams, known as the great foreign *eye doctor,* and one of the grossest impostors, was bound over at Kingston, Upper Canada, a short time since, in the sum of £50, in two securities of £25 each, to appear at the next Court of General Quarter Sessions of the Peace, to answer to a charge of practising physic and surgery without license. The eye-afflicted citizens of Boston will remember this man as long as they retain a recollection of anything—for they were wofully duped. Quite an army might be collected in the different cities, on whose pockets the ex-oculist of the king of the French and the king of Belgium, wrought more tangible effects than on their diseased optics.

[*Boston Med. and Surg. Journal.*

* Alveolar exostosis is by no means unfrequent in its occurrence, and is doubtless the result of inflammation of the alveolar periosteum—favoured by some peculiar constitutional idiosyncrasy. It is an affection in which the gums rarely participate. We have frequently known teeth to be thrusted nearly out of their sockets by it, and at other times to be forced asunder in the manner as described by Mr. Shepherd; the deposition of bone taking place in the one case at the bottom of the alveolus, and in the other only on one of its sides.—*Balt. Ed.*

